# FrzA gene protects cardiomyocytes from H_2_O_2_-induced oxidative stress through restraining the Wnt/Frizzled pathway

**DOI:** 10.1186/s12944-015-0088-0

**Published:** 2015-08-18

**Authors:** Jing Tao, Bang-dang Chen, Yi-tong Ma, Yi-ning Yang, Xiao-mei Li, Xiang Ma, Zi-xiang Yu, Fen Liu, Yang Xiang, You Chen

**Affiliations:** Department of Cardiology, First Affiliated Hospital of Xinjiang Medical University, Urumqi, 830054 P.R. China; Xinjiang Key Laboratory of Cardiovascular Disease Research, Urumqi, 830054 P.R. China

**Keywords:** FrzA, Wnt/frizzled pathway, Oxidative stress, Cardiomyocytes, Apoptosis

## Abstract

**Background:**

Lately, there is accumulating evidence that the Wnt/Frizzled pathway is reactivated after myocardial infarction, the inhibition of the pathway is beneficial since it reduce of myocardial apoptosis and prevents heart failure. FrzA/Sfrp-1, a secreted frizzled-related protein and antagonist for the wnt/frizzled pathway. We assessed the hypothesis that FrzA protects cardiomyocytes from H_2_O_2_-Induced Oxidative damage through the inhibition of Wnt/Frizzled pathway activity.

**Methods:**

We used a recombinant AAV9 vector to deliver FrzA gene into neonatal rat ventricle myocytes and developed an oxidative stress model using H_2_O_2_. The cell vitality was measured by MTT colorimetric assay. Western blot and RT-PCR were used to evaluate the expressions of Dvl-1, β-catenin, c-Myc, Bax and Bcl-2. Flow cytometry analysis of cardiomyocytes apoptosis.

**Results:**

We confirmed that Wnt/frizzled pathway is involved in H_2_O_2_-induced apoptosis in cardiomyocytes. Compared with controls, H_2_O_2_ induced the upregulation of Dvl-1, β-catenin, and c-Myc. FrzA suppressed the expression of Dvl-1, β-catenin, c-Myc and the activity of the Wnt/frizzled pathway. Furthermore, FrzA over-expression decreased the apoptotic rate, and the Bax/Bcl-2 ratio in cardiomyocytes treated with H_2_O_2_.

**Conclusions:**

FrzA, through the inhibition of Wnt/Frizzled pathway activity reduced H_2_O_2_-induced cardiomyocytes apoptosis and could be a potential therapeutic target for prevention of cardiac oxidative damage.

## Introduction

Cardiovascular disease is the leading cause of morbidity and mortality all over the world. Oxidative stress has been implicated in a variety of cardiovascular diseases, including atherosclerosis, hypertension, myocardial infarction, and heart failure [1, 2]. Over-production of oxidative stress attacks the local conformations of DNA, RNA, and proteins in cells [3]. Oxidative stress is a major factor that induces cardiomyocyte apoptosis [4]. However, the mechanisms of oxidative stress in inducing cardiomyocyte apoptosis are poorly understood. Oxidative stress induced myocardial apoptosis cannot be ignored, and new effective therapies are desperately needed.

It is well established that canonical Wnt/frizzled pathway plays a crucial role in regulating numerous cellular processes, including cellular survival, differentiation, proliferation and oncogenesis [5]. Upon Wnt stimulation, the ligand (Wnt) binds to the frizzled receptor and the low-density lipoprotein receptor-related proteins (LRP) co-receptor, the Wnt-frizzled-LRP complex activates the Dishevelled (Dvl) protein which inhibits the activity of GSK3β and leads to cytoplasmic stabilization of bate-catenin (β-catenin). Subsequently, stabilized β-catenin enters the nucleus and activates the transcription of Wnt target genes, such as c-Myc [6]. There is evidence indicating that the aberrant activation of canonical Wnt/frizzled pathway is related to apoptosis in several cell types [7, 8]. Previous study showed that conditional activation of Wnt/frizzled pathway induces a marked increase in the frequency of apoptosis in hematopoietic stem/progenitor cells [9]. Furthermore, knocking down the expression of Dvl-1 partially suppressed the activity of the Wnt/frizzled pathway decreased the apoptotic rate, caspase-3 activity, and the Bax/Bcl-2 ratio in H9C2 cardiomyocytes treated with cyclosporine A [10]. Recently, the role of Wnt/frizzled pathway in cardiac diseases was explored. Wnt/frizzled pathway in the adult heart is quiescent under normal conditions [11], however it is reactivated after injure and in various pathologic states or repair processes [5]. Mice with activated Wnt/frizzled pathway displayed a lower ejection fraction and higher mortality rates [12] while inhibited the activity of the Wnt/frizzled pathway pathway had attenuated cardiac hypertrophy after aortic constriction when compared with wild-type mice [13]. The Wnt/frizzled pathway can be modulated at various levels of this pathway [14] and its inhibition is beneficial since it improves infarct healing and prevents heart failure [15], which leads us to hypothesize that the dysregulation of Wnt/frizzled pathway may be a risk factor of cardiovascular diseases. To the best of our knowledge, it has not been reported that Wnt/frizzled pathway is involved in H_2_O_2_-induced apoptosis in cardiomyocytes.

FrzA/sFRP-1, a secreted frizzled-related protein, possess a cysteine rich domain (CRD) that is similar to a homologous region on the frizzled receptor that binds Wnts [16], and is thought to bind and sequester Wnts away from active receptor complexes. The shared sequence homology between the Frizzled and sFRP CRDs suggests that the binding of Wnt to the sFRP CRD is responsible for the inhibition of Wnt activity by sFRP [17].

The use of adeno-associated virus (AAV) vectors has emerged as a novel method for gene therapy targeting human diseases owing to the nonpathogenic property of these vectors, which transduce both dividing and nondividing cells and support long-term transgene expression [18]. AAV serotype 9 vectors (AVV9) are of particular interest due to their high efficiency of gene transfection in the heart [19]. This study is to investigate the role of inhibition of Wnt/frizzled pathway by AAV9-delivered FrzA in H_2_O_2_-induced apoptosis of cardiomyocytes.

## Materials and methods

### Vectors design

Recombinant AAV9 vectors were purchased from Virovek (Hayward, CA, USA), which were produced with the recombinant baculovirus (rBac)-based system in SF9 cells as described previously[20, 21]. Both recombinant AAV9 vectors were packaged as single-stranded DNA containing enhanced GFP gene (rAAV9-CMV-eGFP, AAV9-eGFP) or FrzA gene (rAAV9-CMV-FrzA, AAV9-FrzA), which was driven by the human cytomegalovirus promoter.

### Isolation, culture of rat cardiomyocytes

The experimental designs and protocols for animal studies were reviewed and approved by Xinjiang management committee for medical laboratory animal sciences. 1-to 3-day-old Sprague–Dawley rats were provided by the Xinjiang Medical University Laboratory Animal Center. Primary neonatal rat cardiac ventricular myocytes (NRVMs) were cultured as previously described [22] with some modifications. Briefly, the hearts of 1-to 3-day-old Sprague–Dawley rats were removed after hypothermia-induced anesthesia by immersion in ice water and were placed in ice-cold 1× Hanks (Sigma, USA). After few rinse, the atria were removed and the ventricles were made two cuts perpendicular to each other (like an open flower) joining at the apex. The tissue was dispersed by digestion with 0.01 % trypsin (Sigma, USA) and 0.08 % collagenase II (Worthington, USA). The cardiacmyocytes were cultivated in DMEM medium supplemented with 10 % FBS (Gibco, USA), 1 % Penicillin-streptomycin (HyClone, USA), 1 % L-Glutamine (Sigma, USA). The compound 5-bromo-29-deoxyuridine (Brdu; 100 μmol/L) was added to inhibit proliferation of fibroblast. The medium was replaced every 48 h.

### Immunofluorescence assay to identify cardiomyocytes

Cardiacmyocytes were cultured for 48 h and fixed in 4 % paraformaldehyde (Shanghai Shenggong, China) for 15 min at room temperature. After washing for 10 min in phosphate-buffered saline (PBS), the cells were permeabilized by 0.25 % Triton X-100 solution (Shanghai Shenggong, China) for 10 min, and then incubated for 30 min in 5 % Donkey serum (ImmunoReagents, USA) to saturate non-specific binding sites. The cells were incubated with a Anti-Cardiac Troponin T antibody (Abcam, USA) (1:200) for 1 h. Then, the cells were washed twice (5 min each) in PBS and incubated in the dark with DyLight 594 conjugated AffiniPure Donkey anti-Mouse IgG(1:200) for 1 h. Finally, the cells were washed twice (5 min each) in PBS and incubated in the dark with 1 μg/ml DAPI (ImmunoReagents, USA) for 1 min. Fluorescent images were documented using a fluorescence microscope (Leica DMI6000B, Germany) at 20× amplification and 10 fields were randomly counted.

### Transduction of primary cardiomyocytes

Cardiomyocytes were seeded into 6-well culture plates in triplicate at a density of 1 × 10^6^ cells per well. Approximately 24 h later, cells were transfected with AAV9-eGFP or AAV9-FrzA virus in serum free medium. The medium containing the viral particles were replaced with the fresh 10 % serum-containing medium 2 h later. The multiplicities of infection (MOI) of AAV9-eGFP and AAV9-FrzA were selected from preliminary experiments (MOI = 6 × 10^5^vg/cell) demonstrating infection efficiency of more than 90 % at the peak time (the peak time at the 5th day was selected from preliminary experiments) [23].

### Measurement of cardiomyocytes vitality

The cell vitality was measured by MTT colorimetric assay as previously described [24]. Briefly, cardiomyocytes cells were seeded in 96 well plates (2 × 10^4^ cells/well), after 48 h seeding, cells were exposed to H_2_O_2_ (30-200 μmol/L) for 6 h, in three parallel wells each, and untreated cells served as a control. Culture supernatant was replaced by complete DMEM, cells were received 25 μL/ well MTT solution (0.2 mg/ml; Sigma) and incubated for 4 h in the dark at 37 °C. The medium was removed and 150 μL DMSO was added to each well. The optical density (OD) was measured using Multiskan GO microplate spectrophotometer (Thermo Fisher Scientific, Waltham, Massachusetts, USA) at a wave length of 490 nm. The cell viability was calculated as a percentage of the control OD.

### Reverse transcription-polymerase chain reaction (RT-PCR) assay

Total RNA was extracted from the cardiomyocytes using RNeasy kit (Qiagene, USA). After DNase treatment, 1 μg total RNA was reverse transcribed for 1 h at 65 °C using 200 units of Superscript III reverse transcriptase (Invitrogen) and 15 ng random primers. The samples were then heated for 15 min at 70 °C. The mRNA levels of Dvl-1, β-catenin and c-Myc were determined by reverse RT-PCR with the G-STORM sequence detection system (Gene Technologies, Somerville, MA, USA). Primers were designed using Primer 5.0 software based on the reported rat sequences and are listed in Table 1 Expression levels were normalized against levels of housekeeping gene β-actin mRNA.

**Table 1:** Primers sequences designed using Primer 5.0 software

Primer name	Primer sequence	Expected size(bp)
Dvl-1	Forward:5'-CAGGGATGCGAGGTTA-3′	130
	Reverse: 5'-AAGGGAGGCAAGAAAGT-3′	
β-catenin	Forward:5'-AAGTTCTTGGCTATTACGACA-3′	375
	Reverse: 5'-ACAGCACCTTCAGCACTCT-3′	
c-Myc	Forward:5'-GCTCTCCGTCCTATGTTGCG-3′	235
	Reverse: 5'-TCGGAGACCAGTTTGGCAG-3′	
β-actin	Forward:5'-GTCCCTCACCCTCCCAAAAG-3′	266
	Reverse: 5'-GCTGCCTCAACACCT-3′	

*Dvl-1* dishevelled-1 protein, *β-catenin* beta-catenin protein, *c-Myc* cellular homologue of avian myelocytomatosis virus oncogene

### Western blot analysis

Western blot analysis was performed according to standard procedures. Equal amounts of protein (40 μg) from each sample were separated on SDS-PAGE precast gels (Invitrogen Life Technologies) and transferred onto nitrocellulose membranes (Invitrogen Life Technologies). After blocking with 5 % skim milk, the membranes were blotted with anti-bodies against β-catenin, c-Myc, Bax, Bcl-2, GADPH (all from Cell Signaling Technology, USA) or Dvl-1(Santa Cruz, USA) by using the Western Breeze chromogenic immunodetection system (Invitrogen Life Technologies),the images were captured and quantified with Image Lab 4.0 software (Bio-Rad Laboratories, USA).

### Flow cytometry analysis of cardiomyocytes apoptosis

Apoptosis was assessed by the Annexin V-FITC Apoptosis Detection kit (KeyGEN) according to the manufacturer’s protocol. Cells were washed with ice-cold PBS, and then resuspended in binding buffer. A total volume of 5 μL Annexin V-FITC stock solution was added and incubated in the dark for 15 min at room temperature. Immediately after mixing with 5 μL propidium iodide solution for another 5 min in the dark, the apoptotic cells were identified by flow cytometry (Beckman Coulter, USA). All the experiments were performed in triplicate.

### Statistical analysis

Statistical analysis was performed with the SPSS (version 19.0; Chicago, IL) software package. All assays were repeated at least three independent times in triplicate. The values for all measurements are presented as the mean ± standard deviation. Comparisons were performed for all pairs using the Student’s t test or, when appropriate, repeated one-way ANOVA for multiple comparisons. The p value less than 0.05 were considered statistically significant.

## Results

### Culture and identification of cardiomyocytes

**Fig. 1:**
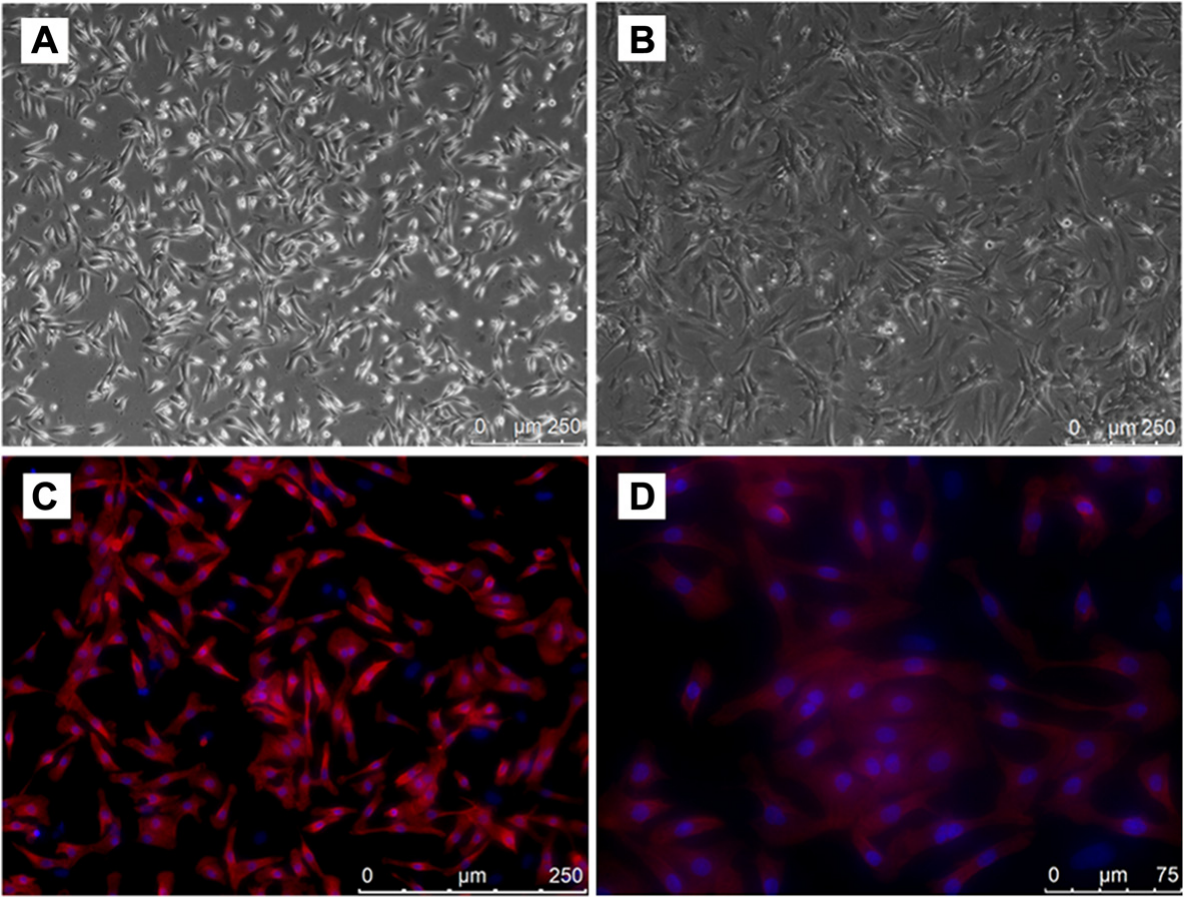
**a** Primary cultured cardiomyocytes for 24 h, cardiomyocytes seen a single pulse; **b** Primary cultured cardiomyocytes for 72 h, seen daisy-shaped formation of cell clusters; **c** Identification results of cardiomyocytes, Immunofluorescence: red for anti-cardiac troponin T antibody specificity stained blue nuclei; **d** Identification results of cardiomyocytes, Imunofluorescence: wire structure is clearly visible

Through improve the conditions and methods of neonatal rat cardiomyocytes in vitro culture, to get high purity, good vigor, complete structure and function of cardiomyocytes. Cardiomyocytes growth of adherent cells after 12 h, 24 h appear individual cells spontaneously beating (Fig. 1a), after 72 h can see daisy-shaped formation of cell clusters (Fig. 1b).

Observed under an inverted fluorescence microscope, red for the anti-cardiac troponin T specific staining, blue is DAPI staining of nuclei, counting, found the purity of myocardial cells can reach more than 98 % (Fig. 1c). At high magnification, the structure of cardiac muscle cell filaments could be clearly observed (Fig. 1d).

### The effect of H_2_O_2_ on cultured cardiomyocyte vitality and apoptosis

**Fig. 2:**
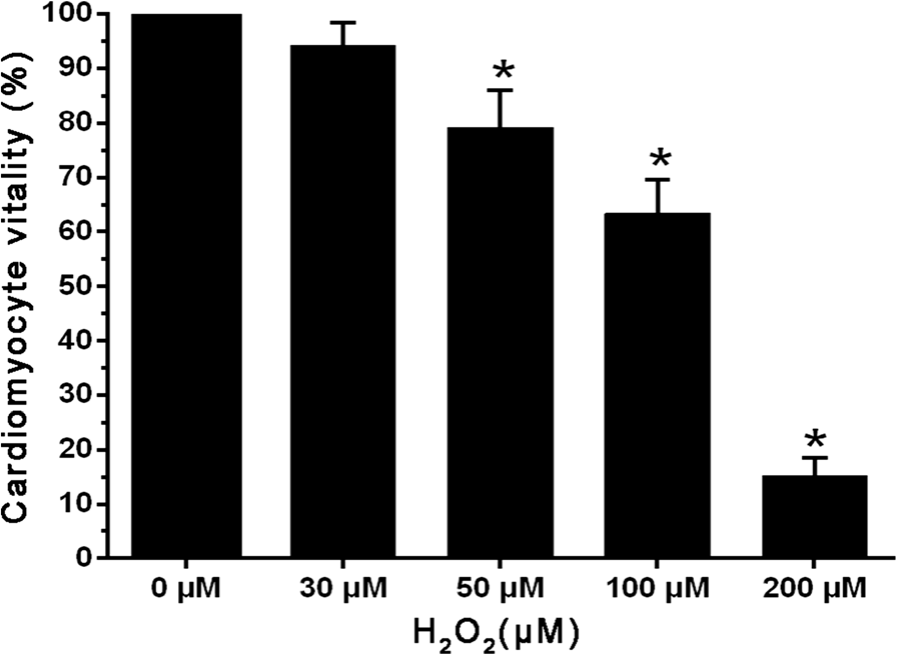
MTT assay cell viability, the cardiomyocytes were incubated with 0,30,50,100 and 200 μM concentrations of H_2_O_2_ for 6 h. The viability of the cardiomyocytes was evaluated using the MTT test. (Note: *Compared with the control group *P* <0.05)

**Fig. 3:**
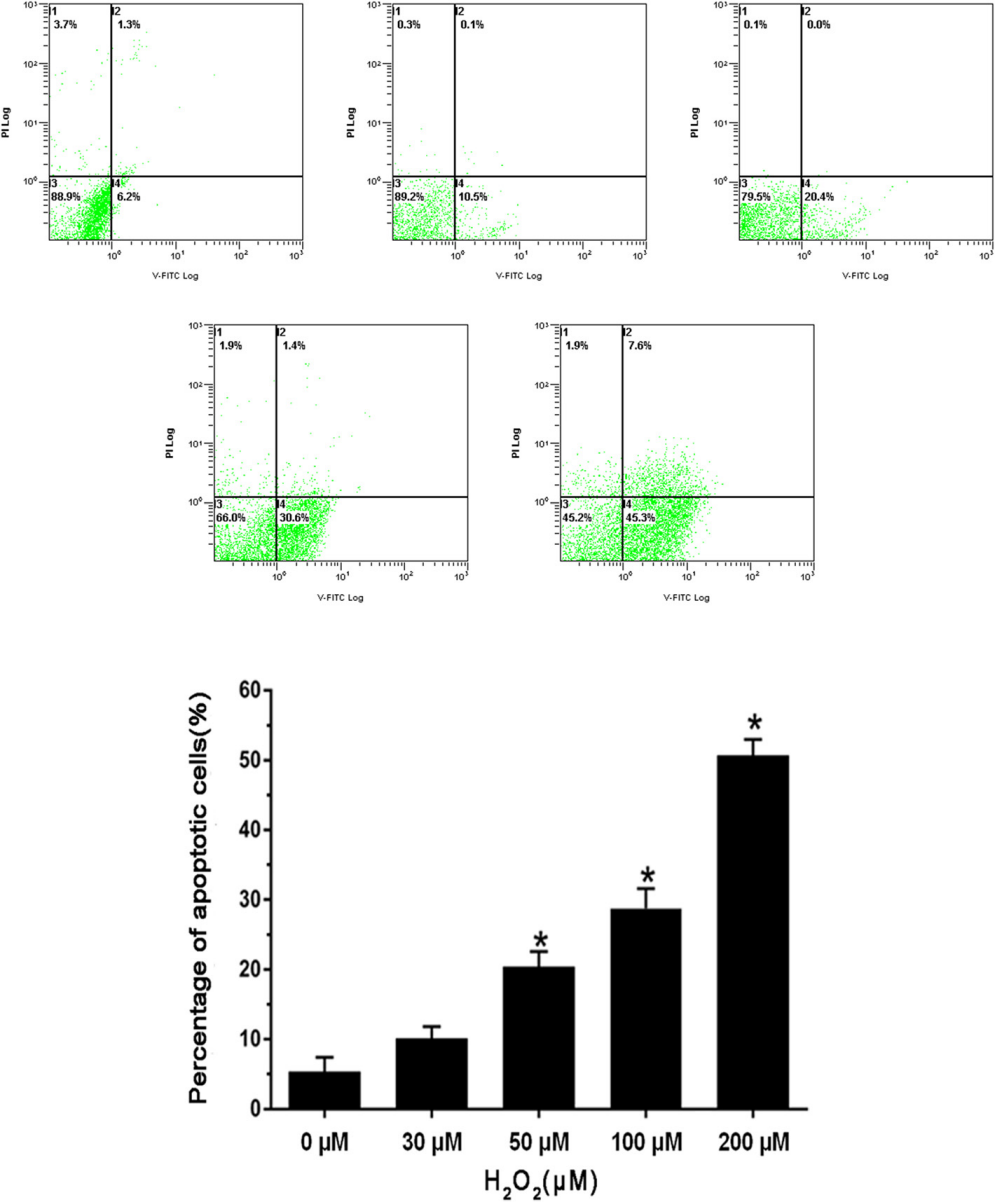
AnnexinV-FITC/PI double staining flow cytometry to detect apoptosis rate, the cardiomyocytes were incubated with 0,30, 50,100 and 200 μM concentrations of H_2_O_2_ for 6 h. The cardiomyocytes apoptosis rate was measured by flow cytometry method. (Note: * Compared with the control group *P* <0.05)

To investigate the effect of H_2_O_2_ on cardiomyocytes vitality and apoptosis, cardiomyocytes were incubated with 30、50、100 and 200 μM concentrations of H_2_O_2_ for 6 h. The viability of the cardiomyocytes was evaluated using the MTT test, we found that the cardiomyocytes viability was significantly decreased in a dose-dependent manner after H_2_O_2_ stimulation compared with the control group (Fig. 2). The apoptosis rate was measured by flow cytometry method, we also found that the cardiomyocytes apoptosis was markedly increased following high concentrations of H_2_O_2_ treatment compared with that control group (Fig. 3). Since 200 μM H_2_O_2_ stimulation induced more than 50 % cells apoptosis and cardiomyocytes vitality was significantly reduced to 12.4 ± 3.7 %. Thus, we have chosen the relative lower concentration of H_2_O_2_ (100 μM), for the further experiment.

### AAV9-FrzA protects cardiomyocytes from H_2_O_2_ induced cytotoxicity

**Fig. 4:**
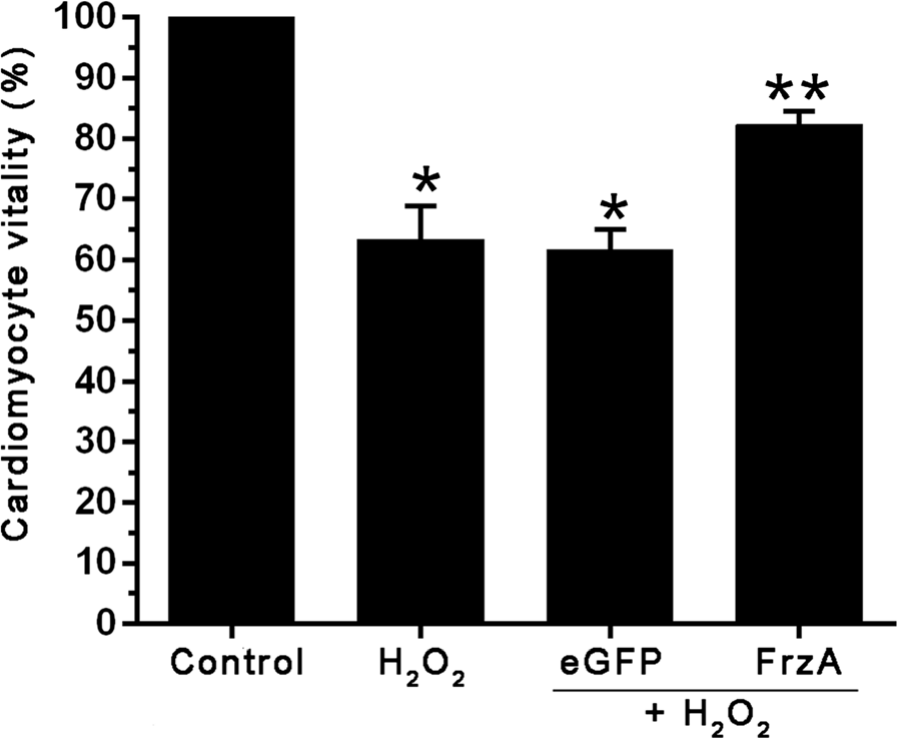
MTT assay cell viability, the cardiomyocytes were pretreated with the indicated concentrations of AAV9-eGFP and AAV9-FrzA for 5 d then further treated with H_2_O_2_ (100 μM), and cell viability measured by the MTT test. (* Compared with the control group *P* <0.05; ** compared with other groups all *P* <0.05)

In order to study whether AAV9-FrzA was able to protect against cell injury induced by oxidative stress in vitro, we determined the H_2_O_2_-induced cardiomyocytes toxity. The cardiomyocytes were pretreated with the indicated concentrations of AAV9-eGFP and AAV9-FrzA for 5 d then further treated with H_2_O_2_ (100 μM), and cell viability measured by the MTT assay(Fig. 4). The cells in control group were considered 100 % viability. Viability of H_2_O_2_ treated cells significantly decreased to 63.3 ± 5.6 % in cell viability. The viability of cells after pretreatment with AAV9- FrzA was around 82.28 %. When the cells were pretreated with AAV9-FrzA, the cell viability increased significantly compared to H_2_O_2_ treated group and the AAV9-eGFP treated group.

### AAV9-FrzA protects cardiomyocytes from H_2_O_2_ induced cell apoptosis

**Fig. 5:**
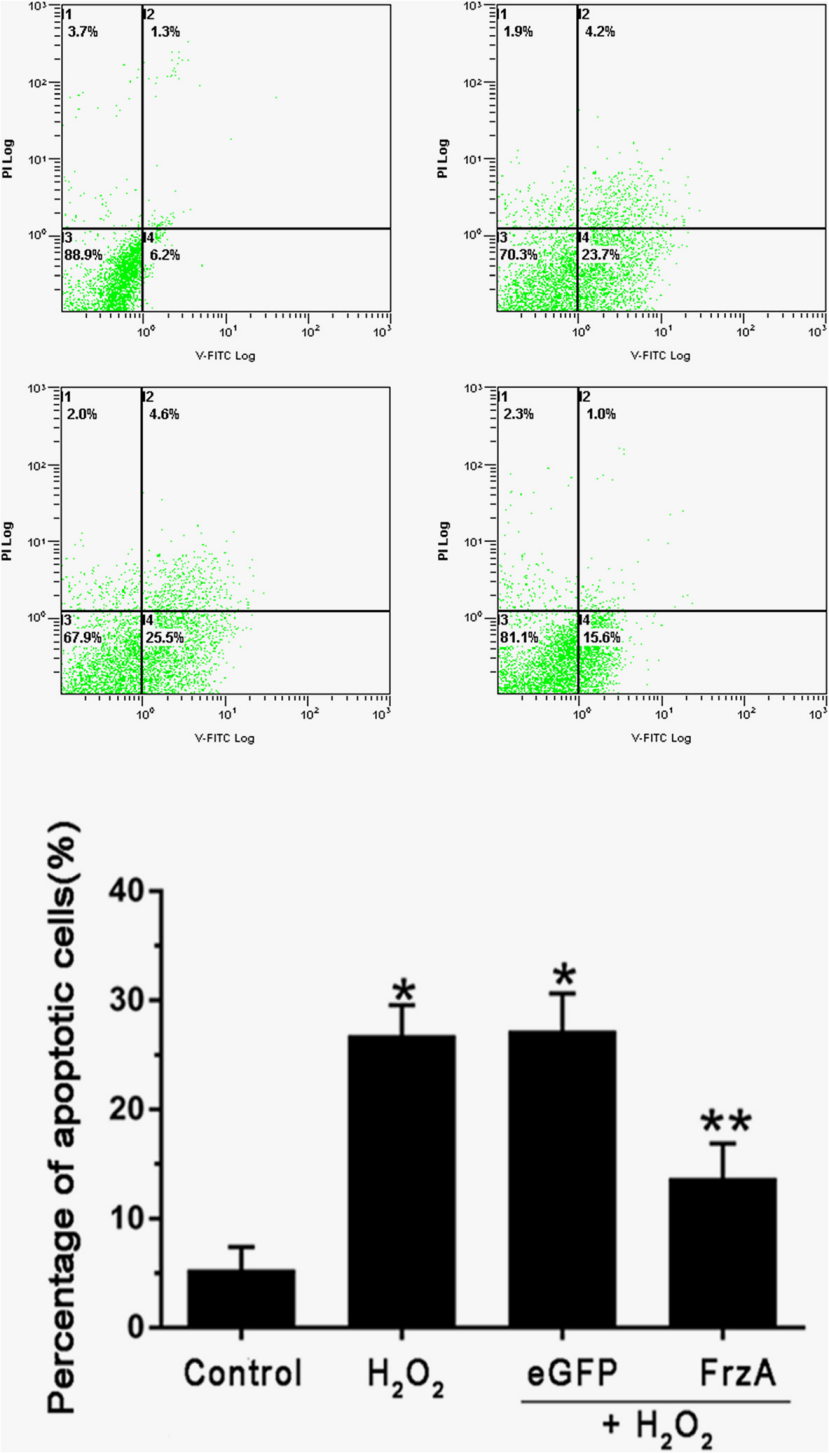
AnnexinV-FITC/PI double staining flow cytometry to detect apoptosis rate, the cardiomyocytes were pretreated with the indicated concentrations of AAV9-eGFP and AAV9-FrzA for 5 d then further treated with H_2_O_2_ (100 μM), and the apoptosis rates were measured by flow cytometry method. (Note: *Compared with the control group *P* <0.05; ** compared with other groups all *P* <0.05)

To confirm the effects of AAV9-FrzA on H_2_O_2_-induced Cardiomyocytes apoptosis, Western blot analysis to examine the expression levels of the Bax, Bcl-2.The apoptosis rates were measured by flow cytometry method. As shown in Fig. 5, the number of apoptotic cells in control group was 6.7 ± 2.1 %. After being treated with 100 μM H_2_O_2_, about 27.2 ± 3.4 % of cells showed apoptosis characteristics. AAV9-FrzA pretreatment decreased the percentage of the apoptotic cells compared with the groups that treated with H_2_O_2_ alone and AAV9-eGFP. These results indicate that AAV9-FrzA can protect cardiomyocytes from H_2_O_2_ induced cell apoptosis.

**Fig. 6:**
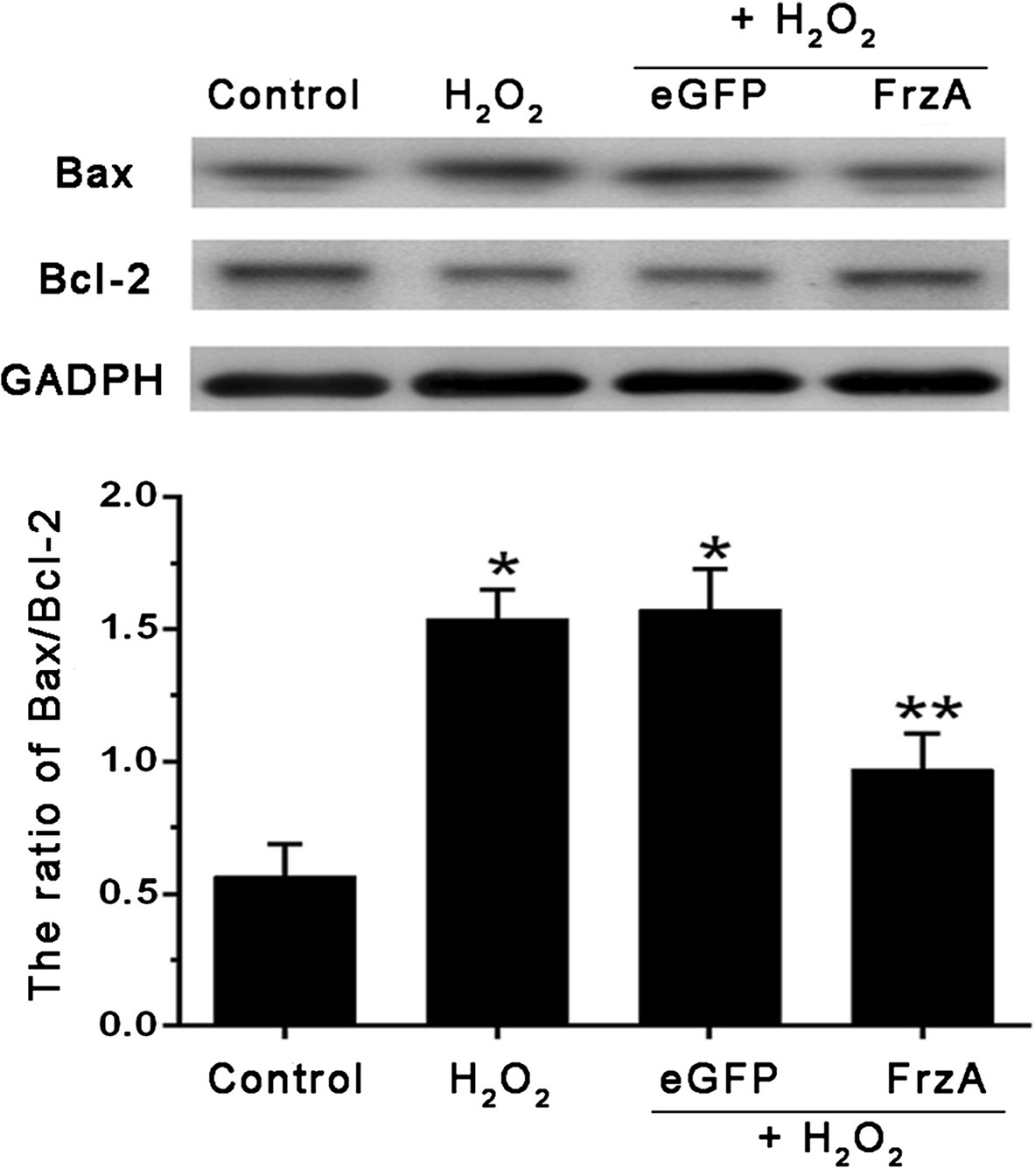
Western Blot detect the expression of apoptosis-related proteins, the cardiomyocytes were pretreated with the indicated concentrations of AAV9-eGFP and AAV9-FrzA for 5 d then further treated with H_2_O_2_ (100 μM), and western blot analysis to examine the expression levels of the Bax, Bcl-2. (Note: * Compared with the control group *P* <0.05; ** compared with other groups all *P* <0.05)

Compared with control group, the levels of Bax/Bcl-2 increased in the H_2_O_2_ group, indicating H_2_O_2_ induced the cardiomyocytes apoptosis. However, compared with H_2_O_2_ group, such high levels of Bax/Bcl-2 was completely abolished by treatment with FrzA infection (Fig.6).

### H_2_O_2_-induced Wnt/frizzled pathway activation in cardiomyocytes was inhibited by AAV9-FrzA infection

**Fig. 7:**
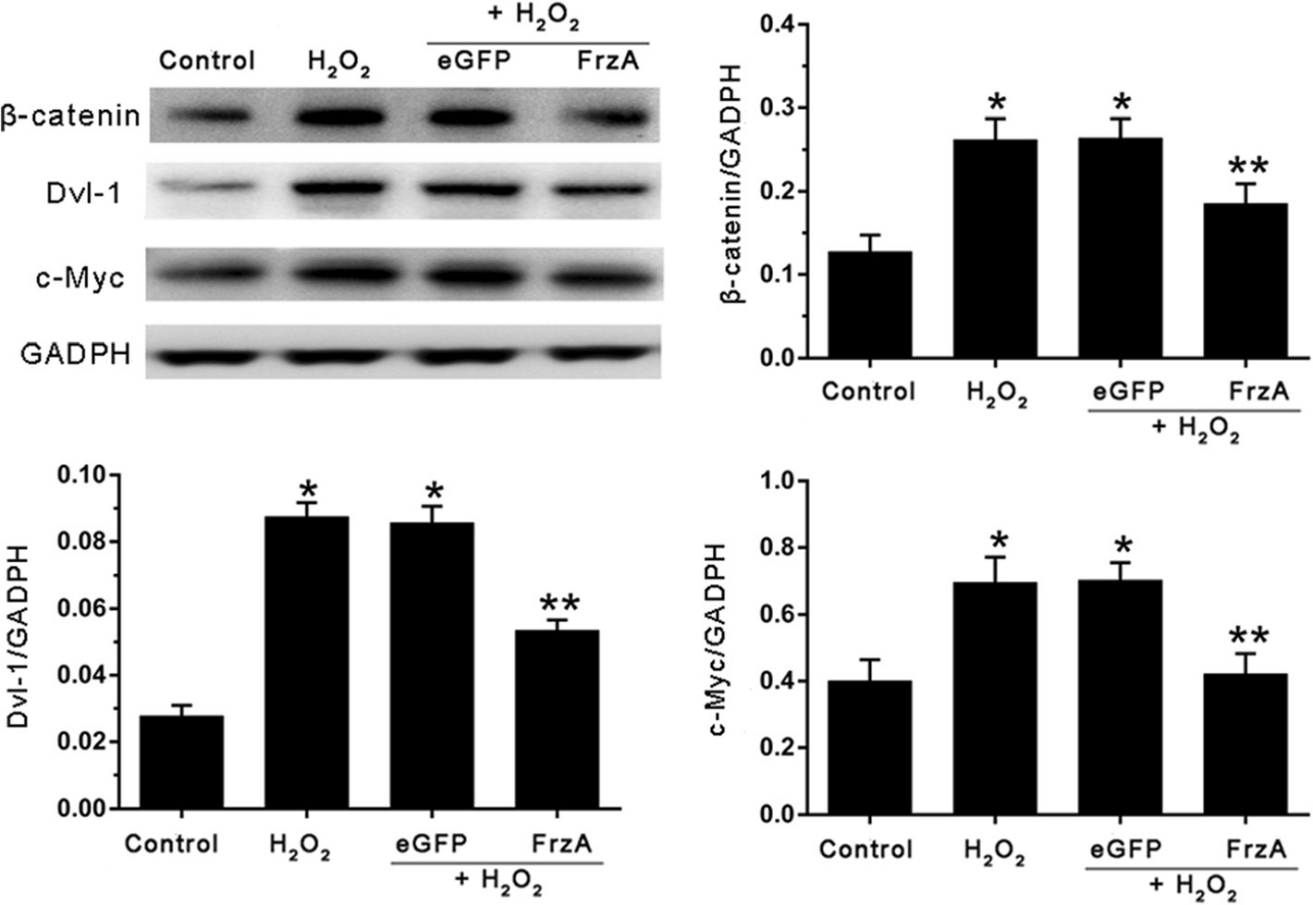
Western blot detect the expression of Wnt/frizzled pathway key molecules, the cardiomyocytes were pretreated with the indicated concentrations of AAV9-eGFP and AAV9-FrzA for 5 d then further treated with H_2_O_2_ (100 μM), and western blot analysis to examine the protein expression levels of the Dvl-1, β-catenin and c-Myc. (Note: * Compared with the control group *P* <0.05; ** compared with other groups all *P* <0.05)

**Fig. 8:**
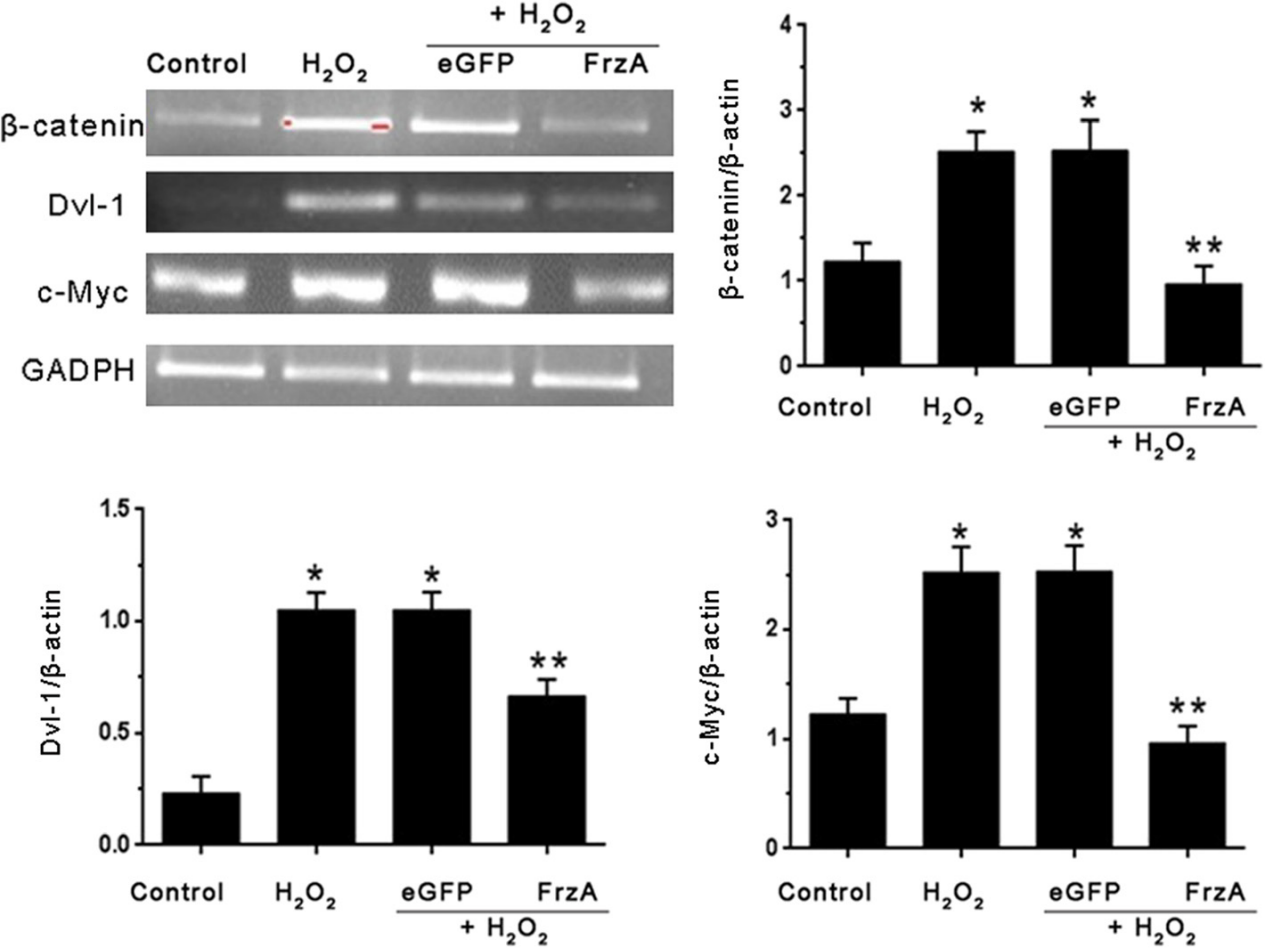
RT-PCR detect the expression of Wnt/frizzled pathway key molecules, the cardiomyocytes were pretreated with the indicated concentrations of AAV9-eGFP and AAV9-FrzA for 5 d then further treated with H_2_O_2_ (100 μM), and RT-PCR analysis to examine the mRNA expression levels of the Dvl-1, β-catenin and c-Myc. (Note: * Compared with the control group *P* <0.05; ** compared with other groups all *P* <0.05)

To investigate whether AAV9-FrzA could inhibite the Wnt/frizzled pathway , western blot and RT-PCR analysis were performed in cardiomyocytes to examine the expression levels of the Dvl-1, β-catenin and c-Myc, which were main molecules about Wnt/frizzled pathway. As shown in Fig. 7, H_2_O_2_ alone, pretreatment with AAV9-eGFP and AAV9-FrzA group resulted in an increase in the protein levels of the Dvl-1, β-catenin and c-Myc compared with the control cells. However, pretreatment with AAV9-FrzA, the expression of Dvl-1, β-catenin and c-Myc decrease compared with the H_2_O_2_ alone cells, *P* < 0.05. In Fig. 8, we found the same results, H_2_O_2_ group had elevated dvl-1, β-catenin, and c-myc mRNA levels, which was efficiently suppressed by FrzA.

## Discussion

H_2_O_2_ treatment resulted in the increased expression of Dvl-1, β-catenin, and c-Myc and increased apoptosis and vitality in cardiomyocytes. Those changes were abrogated by AAV9 mediated FrzA gene transfer.

H_2_O_2_ is one of reactive oxygen species(ROS) and has been widely used in the experiment to mimic situation with oxidative stress. However, the concentration of H_2_O_2_ used in experiments differs widely in different cell types. Different type of cells showed different response to oxidative stress induced by H_2_O_2_ [25]. In this study, we evaluated the effect of H_2_O_2_ on cardiomyocytes and found that cardiomyocytes damage induced by H_2_O_2_ was almost dose-dependent. Also, we have chosen the relative lower concentration of H_2_O_2_ (<100 μM) for further experiment. Similar idea has also been described by Tanaka and Aikawa [26, 27]. Recent study showed that Wnt/Frizzled signaling pathway is activated in the rat hearts after acute myocardial infarction [28]. In our study, we found that Wnt/Frizzled signaling pathway activation is sensitive to H_2_O_2_ stimulation in cardiomyocytes. After H_2_O_2_ treatment, the level of Dvl-1, β-catenin, and c-Myc, which were major members of canonical Wnt/frizzled pathway, were all upregulated.

In recent years, the intervention of the Wnt/frizzled pathway for cardiovascular disease has received increasing attention. These studies investigated the activity of the Wnt/frizzled pathway from different stages. Studies have shown that Dvl-1 is essential for maintaining the integrity of the infracted heart. By knockout Dvl-1 gene, ANF and BNP expression decreased, AKT activity was inhibited, but GSK-3β activity was activated, which relieved cardiac hypertrophy caused by overload pressure [29]. However, in previous studies, transgenic or gene knockout method interferes with Wnt/frizzled pathway in all organizations and cells, resulting in higher embryo mortality [30] and cardiac rupture after myocardial infarction rate [31].β-catenin also plays a very important role in the embryonic development, cell adhesion, and normal cell structure maintenance. Therefore, Dvl-1 and β-catenin are not suitable as an intervention target of Wnt/frizzled pathway.

FrzA, also called sFRP-1, has become a focus of research in recent years as it can negatively regulate the Wnt/frizzled pathway. In FrzA transgenic mice, cardiac rupture rate was reduced from 26.4 % to 6.5 % and myocardial infarct size became smaller after myocardial infarction while the apoptotic index, early leukocyte infiltration, and MMP-2 and MMP-9 activity decreased, collagen distribution wass significantly improved and cardiac function also improved [15]. This protective effect is likely due to inhibition of over-activation of the Wnt/frizzled pathway achieved by sFRP-1 but needs further experimental confirmation [32, 33]. In our study, the expression levels of the Dvl-1, β-catenin, and c-Myc all increased when cardiomyocytes exposed to H_2_O_2_, indicating an activation of the Wnt/frizzled pathway by H_2_O_2_. However, the enhanced expression of Dvl-1, β-catenin and c-Myc were completely abolished by FrzA.

Evidence from this study supports our hypothesis that Wnt/frizzled pathway might be involved in H_2_O_2_-induced apoptosis of cardiomyocytes. Inhibition of Wnt/frizzled pathway by sFRP1 in H_2_O_2_-treated cardiomyocytes resulted in a lower apoptotic rate which was accompanied by the decreases of Bax/Bcl-2 ratio. Bax is a cardinal pro-apoptotic member and Bcl-2 is a pro-survival protein, which can form homodimers or heterodimers with each other, and the ratio between the two proteins determines whether the cell survives or undergoes apoptosis [34]. Increased Bax expression in the presence of reduced Bcl-2 expression likely generates a dominant signal in favor of cell death [35]. The results from current study indicate that Wnt/frizzled pathway may play a critical role in cardiomyocytes apoptosis triggered by H_2_O_2_ and inhibition of canonical Wnt/frizzled pathway conferring cytoprotection of cardiomyocytes, suggesting a therapeutic potential for a variety of heart diseases related to ROS.

## Conclusion

Our study is the first to demonstrate that FrzA, through the inhibition of Wnt/Frizzled pathway activity, reduced H_2_O_2_-induced cardiomyocytes apoptosis and could be a potential therapeutic target to prevent the oxidative damage after cardiovascular infarct.
